# Factors Affecting Patency of Uncovered Duodenal Stents in Malignant Duodenal Stenosis Due to Pancreatic Cancer: A Retrospective Cohort Study

**DOI:** 10.7759/cureus.77245

**Published:** 2025-01-10

**Authors:** Koji Takahashi, Hiroshi Ohyama, Izumi Ohno, Naoya Kato

**Affiliations:** 1 Gastroenterology, Chiba University, Chiba, JPN

**Keywords:** duodenal stenosis, duodenal stent, pancreatic cancer, patency, retrospective cohort

## Abstract

Introduction

Malignant duodenal stenosis is a common complication in patients with pancreatic cancer, significantly impairing quality of life by obstructing oral intake. Duodenal stenting has become a preferred palliative intervention, offering minimally invasive symptom relief and allowing for the continuation of systemic chemotherapy. However, factors influencing stent patency remain underexplored. This study aimed to identify clinical, procedural, and tumor-related factors that affect the patency of uncovered duodenal stents in malignant duodenal stenosis caused by pancreatic cancer.

Methods

A retrospective cohort study was conducted at Chiba University Hospital, Chiba, Japan, analyzing data from 53 patients who underwent placement of 22 mm uncovered duodenal stents between June 2016 and December 2023. Eligibility criteria included that the primary tumor had not been resected and that no intestinal reconstruction had been performed. Data on patient demographics, tumor characteristics, procedural details, and outcomes were collected. Univariate and multivariate analyses were performed to evaluate factors influencing stent patency using the Kaplan-Meier method and Cox proportional hazards modeling.

Results

The mean patency duration for uncovered duodenal stents was 474 days, with stent occlusion occurring in 11 (20.8%) patients. Univariate analysis identified prior placement of transpapillary biliary plastic stents as significantly associated with reduced stent patency (p = 0.0057). Multivariate analysis confirmed this as an independent predictor of shorter patency (hazard ratio, 5.75; 95% CI, 1.37-24.22; p = 0.017). Tumor size, chemotherapy administration, and the location of duodenal stenosis were not significantly associated with stent patency.

Conclusions

Prior placement of transpapillary biliary plastic stents significantly reduces the patency of uncovered duodenal stents in patients with malignant duodenal stenosis caused by pancreatic cancer. This underscores the importance of procedural planning, including consideration of alternative biliary drainage methods, to optimize stent performance and improve patient outcomes.

## Introduction

Pancreatic cancer is frequently accompanied by severe complications necessitating palliative interventions, such as malignant biliary obstruction, gastric outlet obstruction (GOO), and tumor-related pain [1]. Among these, GOO significantly compromises patients’ quality of life, often requiring prompt management to restore oral intake and improve nutritional status. Duodenal stenting has emerged as an effective palliative approach for managing GOO, demonstrating high technical success rates, marked improvements in oral intake, and the potential to maintain systemic chemotherapy [2,3]. Despite its substantial benefits, duodenal stenting is not without risks. Rare but noteworthy complications, including aspiration pneumonia, bleeding, and stent migration, have been reported [2,4]. Nevertheless, for patients with pancreatic cancer and GOO who are unsuitable for surgery, duodenal stenting remains the preferred alternative to palliative surgery, given its minimally invasive nature and rapid symptom relief [4]. There is limited research on factors influencing the patency of uncovered duodenal stents placed for pancreatic cancer-related duodenal stenosis. Identifying these factors is essential to optimize stent selection and procedural strategies, thereby enhancing patient outcomes. This study aims to explore determinants affecting the patency duration of 22 mm uncovered duodenal stents in patients with malignant duodenal stenosis caused by pancreatic cancer, particularly in cases where the primary tumor remains unresected.

## Materials and methods

Study design

This retrospective single-center study was conducted at Chiba University Hospital, Chiba, Japan, and included patients who underwent duodenal stenting with uncovered stents (diameter, 22 mm) for malignant duodenal stenosis caused by pancreatic cancer between June 2016 and December 2023. Patients were excluded if they had undergone primary tumor resection, intestinal reconstruction, or if insufficient data on stent placement and subsequent clinical outcomes were available. Eligible patients were identified using medical records, endoscopic findings, and laboratory results. Subsequently, data on these patients, including their post-treatment outcomes, were collected. Factors associated with stent patency were analyzed using univariate and multivariate methods. 

Definitions

Duodenal stent placement was indicated for patients experiencing nausea or vomiting that hindered oral intake or in those with gastric dilation and duodenal stenosis confirmed via computed tomography and gastrointestinal endoscopy. The final decision to perform stent placement was made during a departmental conference. Adverse events were graded using the American Society for Gastrointestinal Endoscopy lexicon [5]. Tumor staging followed the eighth edition of the American Joint Committee on Cancer staging system [6]. Stent obstruction was classified as ingrowth if caused by tumor growth into the stent lumen and as overgrowth if caused by tumor proliferation over the stent edges. Cases with food impaction but no visible stenosis were not considered obstructive.

Techniques

**Figure 1:**
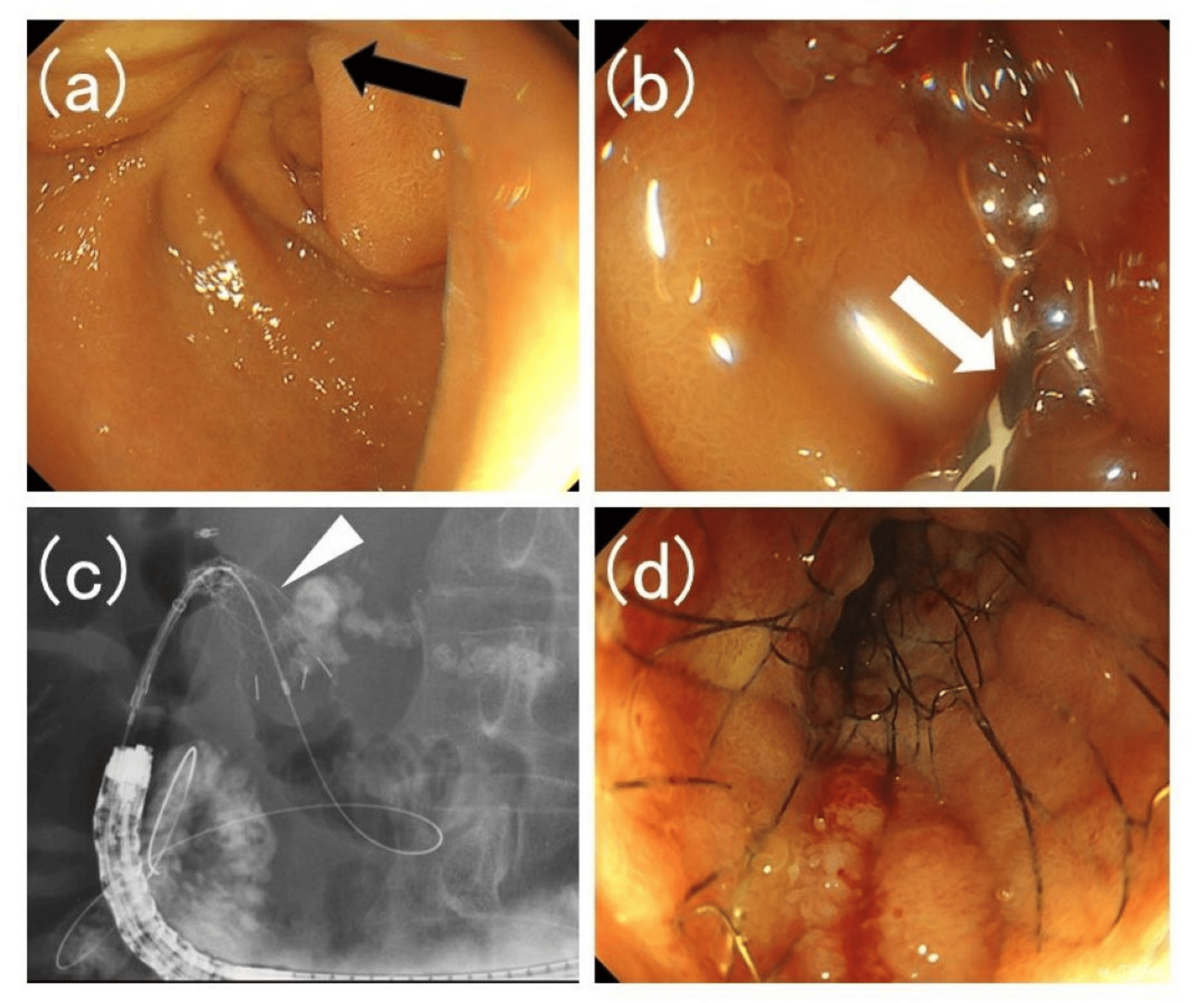
Images of duodenal stent placement (a) An endoscope was advanced to the vicinity of the duodenal stenosis (black arrow). (b) A guidewire (white arrow) was inserted into the duodenal stricture. (c) The duodenal stent delivery system (white arrowhead) was inserted over the guidewire, and deployment was initiated. (d) The duodenal stent was fully deployed.

Stent placement was performed under fluoroscopic guidance using forward-viewing endoscopes (GIF-H290T, PCF-Q260AI, TJF-260V, TJF-Q290V; Olympus, Tokyo, Japan) with carbon dioxide insufflation (Figure 1).

Conscious sedation and intravenous prophylactic antibiotics were administered prior to the procedure. Stents used included Evolution (Cook Medical, Bloomington, IN, USA), HANAROSTENT Naturfit Duo (Boston Scientific, Marlborough, MA, USA), and Niti-S pyloric/duodenal D-type stent (Taewoong Medical, Gimpo, Republic of Korea). All stents had a lumen diameter of 22 mm. The severity of clinical symptoms was evaluated using the obstructive symptoms based on improvements in the Gastric Outlet Obstruction Scoring System (GOOSS) [7].

Statistical analysis

Continuous variables are expressed as means ± standard deviations, while categorical variables are presented as counts and percentages. The Kaplan-Meier method was used to estimate stent patency and survival durations. Univariate analysis for each factor was conducted using the log-rank test. Multivariate analysis was performed with the Cox proportional hazards model, incorporating factors with p-values <0.1 in the univariate analysis. Statistical significance was set at p < 0.05. Cutoff values for continuous variables were defined as the mean of each variable. All analyses were conducted using BellCurve for Excel (Social Survey Research Information Co., Ltd., Tokyo, Japan).

Ethical considerations

This study was approved by the Institutional Review Board of Chiba University Hospital (protocol code: HK202404-01, approval date: April 17, 2024) and adhered to the latest revision of the Declaration of Helsinki. Informed consent was obtained through an opt-out methodology.

## Results

**Figure 2:**
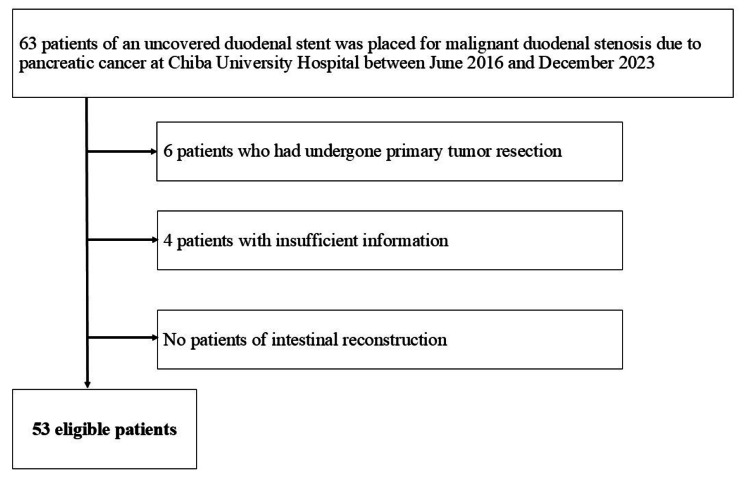
Flowchart of the study population

A total of 63 patients were initially enrolled in this study. After excluding six patients who underwent primary tumor resection and four patients with insufficient data, 53 patients met the inclusion criteria (Figure 2). No cases of intestinal reconstruction were identified.

The background and characteristics of the 53 eligible patients are summarized in Table 1. The mean age was 67.7 ± 10.7 years, and 22 (41.5%) patients were male. At the time of duodenal stent placement, 41 (77.4%) patients had stage IV pancreatic cancer, while 11 (20.8%) patients presented with locally advanced stage III disease. One patient with stage II disease declined primary tumor resection. The primary tumor was located in the pancreatic head in 42 (79.2%) patients, with an average tumor size of 44.2 ± 14.9 mm. Post-stenting chemotherapy was administered to 36 (67.9%) patients.

**Table 1:** Background and characteristics of 53 eligible patients SD, standard deviation

Variables	Value
Age, years, mean ± SD	67.7 ± 10.7
Male, n (%)	22 (41.5%)
Clinical stage of pancreatic cancer at the time of duodenal stent placement, n (%)	
Ⅱ	1 (1.9%)
Ⅲ	11 (20.8%)
Ⅳ	41 (77.4%)
Primary location of pancreatic tumor, n (%)	
Head	42 (79.2%)
Body	7 (13.2%)
Tail	4 (7.5%)
Size of primary tumor, mm, mean ± SD	44.2 ± 14.9
Chemotherapy administered after duodenal stent placement, n (%)	36 (67.9%)
Location of duodenal stenosis, n (%)	
Duodenal bulb or descending part	25 (47.2%)
Horizontal part	28 (52.8%)
Biliary procedures, n (%)	
Prior placement of transpapillary biliary plastic stent	6 (11.3%)
Prior placement of transpapillary biliary metal stent	16 (30.2%)
Prior placement of hepaticogastrostomy stent	4 (7.5%)
Transpapillary biliary metal stenting during duodenal stent placement	2 (3.8%)
Hepaticogastrostomy stent placement at the time of duodenal stent placement	3 (5.7%)
Duodenal stent occlusion, n (%)	11 (20.8%)
Ingrowth of tumor	7 (13.2%)
Overgrowth of tumor	4 (7.5%)
SD, standard deviation	

Adverse events related to duodenal stent placement were observed in three (5.7%) patients: acute pancreatitis in one (1.9%) patient, aspiration in one (1.9%) patient, and duodenal perforation in one (1.9%) patient.

Changes in the GOOSS scores are shown in Table 2. Before stent placement, 30 (56.6%) patients had a GOOSS score of 0 or 1, indicating severe obstruction. After stent placement, 28 (93.3%) of these patients showed improvement, achieving a GOOSS score of 2 or higher. The mean GOOSS score significantly improved from 1.26 ± 1.29 to 2.85 ± 0.60 (p < 0.001), reflecting substantial clinical improvement.

**Table 2:** Changes in GOOSS before and after duodenal stent placement GOOSS, Gastric Outlet Obstruction Scoring System; SD, standard deviation

GOOSS	Before duodenal stent placement	After duodenal stent placement	p-value
0	24	2	
1	6	0	
2	8	2	
3	15	49	
Mean ± SD	1.26 ± 1.29	2.85 ± 0.60	< 0.001

**Figure 3:**
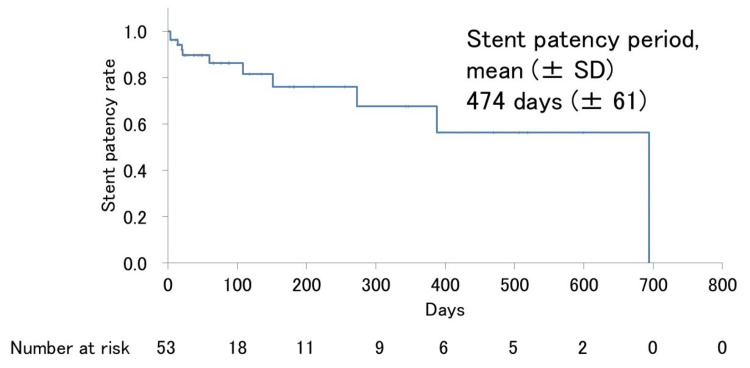
Kaplan-Meier curves for duodenal stent patency The mean duration of stent patency was 474 ± 61 days. SD, standard deviation

The Kaplan-Meier analysis demonstrated a mean duodenal stent patency duration of 474 ± 61 days, with stent occlusion observed in 11 patients (20.8%) (Figure 3). Univariate analysis revealed that the prior placement of transpapillary biliary plastic stents was significantly associated with reduced duodenal stent patency (p = 0.0057). Additionally, while duodenal stenosis located in the bulb or descending portion of the duodenum was not significantly associated with reduced duodenal stent patency (p = 0.076), the p-value was less than 0.1. These two variables were included in the multivariate analysis. The multivariate analysis identified prior biliary plastic stenting as an independent predictor of shorter duodenal stent patency (hazard ratio, 5.75; 95% CI, 1.37-24.22; p = 0.017). Other evaluated factors, including tumor size, chemotherapy administration, and the location of duodenal stenosis, did not demonstrate statistically significant associations with duodenal stent patency (Table 3).

**Table 3:** Univariate and multivariate analyses of factors associated with duodenal stent patency GOOSS, Gastric Outlet Obstruction Scoring System

Variables	Univariate analysis	Multivariate analysis		
	Log-rank test	Cox proportional hazards model		
	p-value	Hazard ratio	95% Confidence interval	p-value
Age (cutoff value: mean, 67.7 years)	0.45			
Male	0.53			
Clinical stage Ⅳ	0.26			
Prior placement of transpapillary biliary plastic stent	0.0057	5.75	1.37-24.22	0.017
Prior placement of transpapillary biliary metal stent	0.32			
Prior placement of hepaticogastrostomy stent	0.28			
Transpapillary biliary metal stenting during duodenal stent placement	0.21			
Hepaticogastrostomy stent placement at the time of duodenal stent placement	0.79			
Primary tumor location in the body or tail	0.38			
GOOSS before stent placement (cutoff value: mean, 1.3)	0.30			
Duodenal stenosis located in the bulb or descending portion of the duodenum	0.076	3.37	0.83-13.69	0.089
Size of primary tumor (cutoff value: mean, 44.2 mm)	0.20			
Chemotherapy after duodenal stent placement	0.51			

## Discussion

The aim of this study was to identify factors influencing the stent patency of a 22 mm uncovered duodenal stent in patients with malignant duodenal stenosis caused by pancreatic cancer. The findings revealed that prior placement of transpapillary biliary plastic stents significantly reduced duodenal stent patency. This effect may be partly attributed to mechanical interference during duodenal stent deployment, which can hinder full expansion and contribute to premature dysfunction. The observed reduction in stent patency underscores the importance of meticulous procedural planning. In cases of duodenal stenosis, endoscopic ultrasound-guided hepaticogastrostomy may be a more suitable option for biliary drainage, as it minimizes interference during stent placement.

Additionally, the GOOSS score in our study showed significant improvement following duodenal stent placement. However, in some cases, the score did not improve, potentially due to intestinal stenosis at multiple sites caused by peritoneal dissemination or diminished intestinal peristalsis.

Duodenal stents have emerged as an effective palliative treatment for malignant gastroduodenal obstruction and offer a safer alternative to surgical intervention [8]. Studies on duodenal stents have demonstrated significant improvements in patients’ ability to tolerate food intake and quality of life following stent placement [9,10]. A multicenter study comparing duodenal stent placement and gastrojejunostomy reported no significant differences in clinical outcomes between gastrojejunostomy and duodenal stent placement for unresectable pancreatic cancer prior to chemotherapy initiation [11]. Common adverse events associated with duodenal stent placement include stent ingrowth/overgrowth, bleeding, and migration; severe adverse events such as perforation are rare [9]. The median duration of duodenal stent patency ranges from six to nine months. Depending on the patients’ condition and cancer type, some patients have survival times exceeding the duration of stent patency after duodenal stent placement [10,12,13]. Stent dysfunction occurs in 7.7-13% of cases, primarily due to tumor growth [12,13]. Patients with duodenal stenosis secondary to pancreatic cancer may exhibit concomitant biliary stenosis. A retrospective study of 100 patients with unresectable pancreatic cancer who underwent endoscopic duodenal stent insertion reported a 91% success rate for combined biliary and duodenal stenting [14].

Although this study did not investigate the relationship between the properties of duodenal stents and their patency, several reports have explored the characteristics of duodenal stents, particularly regarding the presence or absence of a cover and axial force, even in contexts beyond pancreatic cancer. Studies comparing covered and uncovered duodenal stents for malignant gastroduodenal obstruction have reported mixed results. Both types have demonstrated similar technical and clinical success rates. However, covered stents are associated with a higher risk of migration, whereas uncovered stents are more prone to tumor ingrowth [15,16]. Kim et al. reported that uncovered stents may have longer patency durations compared to covered stents [16]. Overall complication rates and patient survival times appear to be similar between the two stent types [15,16]. The choice between covered and uncovered stents may depend on the specific clinical situation, considering the risks of migration against tumor ingrowth [17]. A randomized controlled trial of duodenal stent placement for GOO compared uncovered and partially covered stents and showed that partially covered stents did not improve the frequency of re-intervention or the patency period compared to uncovered stents [18]. Okuwaki et al. investigated the difference in axial force and effectiveness of duodenal stents by randomly assigning 34 patients with pancreatic cancer and GOO to either the high axial force stent group using the WallFlex duodenal stent (Boston Scientific) or the low axial force stent group using the Niti-S pyloric/duodenal D-type stent (Taewoong Medical), and their study revealed no significant difference in clinical effectiveness between the two groups [19].

One of the key strengths of this study was its focus on a well-defined cohort of patients with advanced pancreatic cancer and malignant duodenal stenosis, providing important insights into the relationship between prior biliary procedures during duodenal placement and duodenal stent patency. The use of multivariate analysis to identify significant factors influencing stent patency enhanced the robustness of the findings. Additionally, the study’s retrospective design allowed for the collection of comprehensive clinical data from a single center, providing a detailed understanding of patient outcomes following duodenal stent placement. However, this study had several limitations. First, our study had a relatively small sample size (n = 53) and was conducted at a single center, which may limit the generalizability of the results. Second, the retrospective design has inherent limitations, such as potential biases in data collection and reliance on medical records, which may not capture all relevant variables. Finally, in this study, the cutoff value for the statistical analysis of continuous variables was defined as the overall mean value; however, the way in which this was determined may need to be reconsidered. Future multicenter studies with larger sample sizes should be conducted to validate these findings and further explore the factors affecting stent efficacy in this patient population. Further investigation into different stent types, materials, and techniques, including randomized trials comparing partially covered and newer stents with uncovered stents, could offer insights into re-intervention rates and patency.

## Conclusions

This study identified prior placement of transpapillary biliary plastic stents as a significant factor in reducing the patency of uncovered duodenal stents in patients with malignant duodenal stenosis caused by pancreatic cancer. These findings underscore the importance of procedural planning, particularly in selecting appropriate biliary drainage methods, to optimize stent performance. While duodenal stenting remains an effective palliative option, further studies are needed to evaluate alternative techniques and stent types to improve outcomes in this patient population. Multicenter prospective trials with larger sample sizes are essential to confirm these findings and guide clinical decision-making.
